# Continuous Multi-Parameter Heart Rate Variability Analysis Heralds Onset of Sepsis in Adults

**DOI:** 10.1371/journal.pone.0006642

**Published:** 2009-08-14

**Authors:** Saif Ahmad, Tim Ramsay, Lothar Huebsch, Sarah Flanagan, Sheryl McDiarmid, Izmail Batkin, Lauralyn McIntyre, Sudhir R. Sundaresan, Donna E. Maziak, Farid M. Shamji, Paul Hebert, Dean Fergusson, Alan Tinmouth, Andrew J. E. Seely

**Affiliations:** 1 Ottawa Hospital Research Institute, Ottawa, Ontario, Canada; 2 Division of Thoracic Surgery, University of Ottawa, Ottawa, Ontario, Canada; 3 Department of Clinical Hematology, University of Ottawa, Ottawa, Ontario, Canada; 4 Department of Critical Care Medicine, University of Ottawa, Ottawa, Ontario, Canada; University of East Piedmont, Italy

## Abstract

**Background:**

Early diagnosis of sepsis enables timely resuscitation and antibiotics and prevents subsequent morbidity and mortality. Clinical approaches relying on point-in-time analysis of vital signs or lab values are often insensitive, non-specific and late diagnostic markers of sepsis. Exploring otherwise hidden information within intervals-in-time, heart rate variability (HRV) has been documented to be both altered in the presence of sepsis, and correlated with its severity. We hypothesized that by continuously tracking individual patient HRV over time in patients as they develop sepsis, we would demonstrate reduced HRV in association with the onset of sepsis.

**Methodology/Principal Findings:**

We monitored heart rate continuously in adult bone marrow transplant (BMT) patients (n = 21) beginning a day before their BMT and continuing until recovery or withdrawal (12±4 days). We characterized HRV continuously over time with a panel of time, frequency, complexity, and scale-invariant domain techniques. We defined baseline HRV as mean variability for the first 24 h of monitoring and studied individual and population average percentage change (from baseline) over time in diverse HRV metrics, in comparison with the time of clinical diagnosis and treatment of sepsis (defined as systemic inflammatory response syndrome along with clinically suspected infection requiring treatment). Of the 21 patients enrolled, 4 patients withdrew, leaving 17 patients who completed the study. Fourteen patients developed sepsis requiring antibiotic therapy, whereas 3 did not. On average, for 12 out of 14 infected patients, a significant (25%) reduction prior to the clinical diagnosis and treatment of sepsis was observed in standard deviation, root mean square successive difference, sample and multiscale entropy, fast Fourier transform, detrended fluctuation analysis, and wavelet variability metrics. For infected patients (n = 14), wavelet HRV demonstrated a 25% drop from baseline 35 h prior to sepsis on average. For 3 out of 3 non-infected patients, all measures, except root mean square successive difference and entropy, showed no significant reduction. Significant correlation was present amongst these HRV metrics for the entire population.

**Conclusions/Significance:**

Continuous HRV monitoring is feasible in ambulatory patients, demonstrates significant HRV alteration in individual patients in association with, and prior to clinical diagnosis and treatment of sepsis, and merits further investigation as a means of providing early warning of sepsis.

## Introduction

Severe sepsis and septic shock are major causes of mortality and costs in critically ill patients [1]–[2]. Acute neutropenia is a frequent and intended iatrogenic side effect of cytotoxic chemotherapy and radiotherapy, commonly employed in the management of malignant hematological diseases, most commonly leukemia and lymphoma, leading to increasing risk of opportunistic infections and sepsis [3]. This is particularly apparent in individuals undergoing induction chemotherapy and bone marrow transplantation (BMT) for acute leukemia due to severe prolonged neutropenia [4]. Thus, patients undergoing bone marrow transplantation comprise a group which is at a high risk of systemic infection (approximately 80%) and mortality (approximately 5%) [5]–[6].

Results of a large single-centre randomized trial [7]–[8] indicate that, in early septic shock, mortality can be greatly reduced by employing early goal directed therapy (EGT). In addition, early effective antibiotic therapy is essential to minimize mortality secondary to septic shock [9]. Thus, early diagnosis of sepsis leading to aggressive resuscitation involving antibiotic administration, and maintaining adequate systemic oxygen delivery and tissue perfusion is vital to survival. Current clinical approaches for diagnosing sepsis are based on an increased absolute value of one or more vital signs in addition to laboratory tests such as blood cultures to look for evidence of a pathogen. Although this is the best clinical approach currently available, it represents a crude and potentially non-sensitive and non-specific means for early diagnosis of sepsis.

An alternative approach is to consider the host response to sepsis as a complex system, and utilize variability analysis as a means to better characterize the system [10]. Evidence from experimental and clinical studies indicates that heart rate variability (HRV) is altered in the presence of systemic infection [11]–[13] and correlates with its severity [14]–[15]. Investigators studying the prognostic value of HRV have shown it to be an early predictor of death [16] and multiple organ dysfunction syndrome (MODS) [17] in adult septic patients. Studies on infants and neonates have shown HRV to be predictive [18]–[19] and diagnostic [20] of sepsis.

For adult patients, prior studies have only assessed HRV for short intermittent epochs of 5–15 minutes. To our knowledge, there have been no published attempts to accomplish continuous HRV analysis in adult patients at high risk of sepsis, and no comprehensive analysis of HRV has been performed during the process of development of sepsis. The method that we propose examines a panel of HRV metrics computed continuously over time prior to and during the clinical diagnosis of sepsis (defined as systemic inflammatory response syndrome along with clinically suspected infection requiring treatment) in patients at high risk for systemic infection, namely neutropenic patients following bone marrow transplant. In this pilot study, we demonstrate that (a) it is feasible to enroll and perform continuous HRV analysis of prospectively collected non-stationary heart rate (HR) data in ambulatory patients, (b) continuous variability analysis demonstrates a reduction in individual patient HRV in association with the presence of sepsis, and (c) the alteration in HRV occurs within a clinically relevant period prior to when standard clinical measures lead to clinical diagnosis and treatment.

## Methods

### Ethics Statement

Written informed consent was obtained from all participants and the Ottawa Hospital Research Ethics Board authorized the study.

### Design

This is a descriptive study in which prospective continuous HR recording, and retrospective analysis of both HRV and change in HRV were analyzed over time in ambulatory outpatients as they underwent BMT, and developed neutropenia associated with high risk for developing systemic infection.

### Participants

Our subjects included patients (n = 21) undergoing BMT for hematological malignancy or other disorders (see Table 1) at the Ottawa Hospital (General Campus), Ontario, Canada. All patients were enrolled between 05/2007 to 05/2008. Inclusion criteria included treatment with myeloablative chemoradiotherapy followed by an allogeneic or autologous BMT, and informed consent. Exclusion criteria were pre-existing cardiopulmonary disease, taking beta-blockers or calcium-channel blockers, pre-existing arrhythmia (e.g. atrial fibrillation, atrial bigeminy), contraindication to electrocardiogram adhesives (e.g. allergy, severe psoriasis), and not being fluent in English or French (which precluded patients from understanding and signing the informed consent).

**Table 1:** Study and patient characteristics, along with reason and type of BMT.

	Ottawa (n = 17)
**Study characteristics**	
Follow-up (days), median (IQR)	12(9–14)
Clinical diagnosis of sepsis	14(82%)
Admitted to ICU	0
Deaths	0
**Patient characteristics**	
Age (years), median (IQR)	51(46–62)
Women	5(29%)
Diabetes mellitus	1(6%)
History of heart disease	0
**Reason for BMT**	
Chronic idiopathic myelofibrosis	1(6%)
Crohn's disease	1(6%)
Myelodysplastic syndrome	1(6%)
Non-hematologic malignancy	1(6%)
Leukemia	3(18%)
Myeloma	4(24%)
Lymphoma	6(35%)
**Type of BMT**	
Allogeneic Transport BMT	4(24%)
Autologous BMT	13(76%)

Data are number (%) unless otherwise stated.

### Heart Rate (HR) Monitoring

We collected continuous Holter ECG data (average 12 [SD 4] days of HR monitoring) for all patients in the study, starting approximately 24 h before their BMT and continuing through neutropenia until its resolution or until withdrawal from the study. 3M Cavilon spray combined with daily replacement and relocation of gel ECG electrodes was utilized to reduce any skin irritation. We used a Zymed DigiTrak-Plus Holter system (Philips Healthcare, Markham, Ontario, Canada), which annotated all normal QRS peaks and arrhythmias, including premature atrial and ventricular beats. Only the beats that characterized normal sinus rhythm (NSR) were included, while all premature beats were excluded. RR intervals were derived from R wave annotations. Thus, for each patient, the input to our signal processing algorithms comprised time-stamped NSR-based RR intervals in seconds.

### Feasibility

The feasibility measures evaluated included the number of patients dropping out of the study, the ability of patients to maintain a diary of clinical events (for example, temperature, diarrhea, vomiting, etc.), and compliance with Holter HR monitoring during enrollment. A patient was considered to be a dropout if he or she enrolled in the study, and subsequently discontinued Holter HR monitoring (i.e. due to discomfort, or other factors). A minimum of 72 h of monitoring was necessary to include patients in the study. We examined patient diaries for completeness and regularity in reporting clinical events as per given schedule and instructions. To quantify compliance with monitoring, we computed a ratio between the total time for which patients were monitored and the total time of lost data during the monitoring period. More specifically, percentage of data lost was computed as D_L_ = 100*[T_L_/(T_E_−T_S_)], where D_L_ is percentage of data lost, T_S_ is monitoring start time, T_E_ is monitoring end time, and T_L_ is total time of lost data between T_S_ and T_E_.

### Diagnosis of Sepsis

In this study, sepsis was defined as systemic inflammatory response syndrome along with clinically suspected infection requiring treatment. Patients who were administered broad spectrum antibiotics by the physician (attending hematologist) were considered to have been clinically diagnosed with sepsis. Since exact time of diagnosis was uncertain, we assumed the time (hour and minute) of first administration of antibiotics as the time of clinical diagnosis of sepsis for consistency in procedure. It was not possible to identify precisely the actual time of physician diagnosis. Review of cases and procedures indicated that the diagnosis preceded the first administration of antibiotics by 30 to 90 minutes. The clinical indication to treat sepsis and the clinical diagnosis of sepsis are included in Table 2. Over 50% of the patients had sepsis diagnosed based on the presence of fever, defined *a priori* as one recording greater than 38.5 degrees centigrade or two recordings greater than 38.0 degrees centigrade within 12 h.

**Table 2:** Indication for antibiotics and bacteriological diagnosis.

	Ottawa (n = 14)
**Clinical indication for antibiotics**	
Bacteremia	1(7%)
Productive cough	2(14%)
Mucositis	2(14%)
Clinical suspicion	5(36%)
Fever	7(50%)
**Bacteriological diagnosis**	
Escherichia coli	1(7%)
Streptococcus salivarius	1(7%)
Staphylococcus aureu	1(7%)
Klebsiella pneumoniae	2(14%)
Viridans group streptococcus	2(14%)
Unknown	9(64%)

Data are number (%) unless otherwise stated.

### Variability Analysis

We have developed a novel system for continuous individualized multiorgan variability analysis (CIMVA), with single-organ (the heart) application in this study. The CIMVA system (developed in Windows^®^ Matlab^®^) comprises algorithms for computing and visualizing diverse measures of HRV. Details of diverse HRV measures computed by CIMVA are provided elsewhere [21], but in Table 3 we present a brief summary.

**Table 3:** Summary of signal analysis capabilities of CIMVA.

	Summary
**Time Domain**	
Standard Deviation (SD)	Computed as SD = SQRT [(1/N)*SUM(RR_i_-M)^2^], i = 1 to N, where RR_i_ is i^th^ of N inter-beat intervals and M is their mean. Measures signal variability from its mean value.
Root Mean Square Successive Difference (RMSSD)	Computed as RMSSD = SQRT[{1/(N-1)}*SUM(RR_i_-RR_i-1_)^2^], i = 2 to N, where RR_i_ is i^th^ of N inter-beat intervals. Measures variability of successive signal values.
**Complexity Domain**	
Sample Entropy (SampEn)	Computed as negative logarithm of estimate of conditional probability that RR interval epochs of length m that match pointwise within tolerance r also match at the next point. Characterizes “meaningful structural richness”, information, or disorder of signal.
Multiscale Entropy (MSE)	Measures SampEn on multiple timescales. Multiscaling is achieved by averaging non-overlapping samples. Accounts for dependence of entropy measures on timescale.
**Frequency/Time-Frequency Domain**	
Fast Fourier Transform (FFT)	Computed by transforming RR interval signal to frequency domain. AUC of bands (HF: 0.18–0.4 Hz, LF: 0.04–0.15 Hz) in power spectrum plot characterizes signal variability.
Maximal Overlap Discrete Wavelet Transform (MODWT)	Computed by transforming RR interval signal to time-frequency domain by convolving it with least asymmetric 8-tap (LA8) wavelet filter. AUC of spectral density plot characterizes variability (fluctuations) in time and frequency simultaneously.
**Fractal Domain**	
Detrended Fluctuation Analysis (DFA)	Computed as overall root-mean-square fluctuation F(n) of integrated and detrended RR signal on multiple timescales n. Linear log-log plot of F(n) versus n indicates fractal scaling and AUC (or intercept) and slope characterizes variability.
Power Law Analysis (PLA)	Computed as frequency distribution of squared difference between RR signal and its mean. Linear log-log plot of frequency versus value indicates fractal scaling and intercept and slope characterizes variability.

To accomplish continuous variability analysis over time, CIMVA employs a *moving window* approach, whereby a window (interval-in-time) of user specified *width* and *step* marches through the input signal, computing and time-stamping different variability metrics at each *step*, thus making it possible to monitor a change in HRV over time. A standard RR cleaning algorithm [22] is employed inside each window to detect gross artifact or noise, and HRV analysis is performed on the cleaned data. The cleaning algorithm excludes RR intervals less than 0.25 s and greater than 2.5 s, as well as those that differ by more than 15% from the previous one. The CIMVA system stores the number of samples lost due to RR cleaning in each window instance, thus keeping track of signal quality.

We employed a window *width* of 1200 samples (∼10 minutes) and *step* of 200 samples (∼2 minutes) to compute HRV over time. In Table 4, we present an example of the output from the CIMVA system.

**Table 4:** Example of first five window instances of a CIMVA generated change in HRV over time matrix for diverse measures for a moving window *width* of 1200 samples and *step* of 200 samples.

Window Start Time	Window End Time	Window Start Index	Window End Index	Samples Lost to RR Cleaning	SD	RMSSD	Power Law Slope	Power Law Intercept	SampEn	MSE AUC	FFT LF/HF	FFT LF	FFT HF	DFA AUC	DFA Alpha	Wavelet AUC
15∶19	15:30	1	1200	0	0.04	0.011	−1.21	−4.75	0.63	1.42	3.94	7E-04	2E-04	−2.52	1.47	−37.01
15:21	15:31	201	1400	0	0.03	0.011	−1.17	−5.04	1.49	2.41	4.05	6E-04	1E-04	−2.75	1.37	−38.27
15:23	15:33	401	1600	0	0.03	0.012	−1.21	−5.13	1.60	2.76	4.21	6E-04	1E-04	−2.69	1.37	−37.54
15:25	15:35	601	1800	0	0.03	0.012	−1.10	−4.70	1.58	2.69	4.04	7E-04	2E-04	−2.63	1.41	−36.82
15:26	15:37	801	2000	0	0.04	0.012	−0.88	−3.83	0.69	1.62	4.01	8E-04	2E-04	−2.64	1.45	−37.88

Subsequently, the variability time series (HRV over time) underwent a second data integration step or “smoothing” process. That is, the change in HRV over time was smoothed utilizing a moving average and hourly data interpolation technique. This smoothing approach stabilized the change in variability over time to characterize long-term behavior of various variability metrics and facilitated the study of lead times (ΔT_P_) for percentage changes (ΔHRV) from baseline HRV.

### Continuous Analysis

We defined baseline variability as the mean variability for the first 24 h of recording, prior to transplantation. Given expected variation in baseline variability from patient to patient, for all measures of HRV, we computed an *individualized percentage change in variability* with respect to the baseline variability (ΔHRV = 100 * [baseline-current]/baseline). We studied the extent and timing of ΔHRV achieved by various metrics for infected and non-infected patients. To this end, we evaluated individual and between-patient average ΔHRV data. We defined *a priori* a significant ΔHRV as a 25% loss of variability. We also computed mean population correlation amongst all diverse HRV measures for the entire monitoring period.

## Results

Monitoring was initiated in 21 patients. Four patients dropped out within 24 h of initiation of monitoring due to discomfort or other reasons, leaving 17 (81%) datasets for analysis. We observed that the requirement for patients to keep a diary of clinical events on a regular basis was difficult. We found general errors in the diaries. These included the inability to record the correct time of the occurrence of an event, and reporting inaccurate frequency ranges of occurrence of events like vomiting and loose motions. For n = 17 patients who completed the study, for a total monitoring time of 188 days, 12 days (6%) of data was lost due to non-compliance to monitoring. The reasons for non-compliance to monitoring included taking off the Holter due to discomfort or in order to take a bath, or arriving late at the hospital which led to a delay in attaching a new Holter after the old one had reached its recording limit. In terms of data quality, for n = 17, for 176 days (monitoring days - lost days of data = 188−12 = 176) of available RR-interval data, the data lost to RR cleaning was 1.06 days (0.6%).

In the course of a median follow-up of 12 (IQR 9-14, SD 4) days for n = 17, we observed that 14 patients were diagnosed and treated for sepsis, whereas 3 were not. All patients recovered; no patient was admitted to the ICU or died. Out of the 14 infected patients, on an average, 12 (86%) showed a significant (25%) drop (ΔHRV) prior to sepsis for standard deviation (SD), root mean square successive difference (RMSSD), sample entropy (SampEn), multiscale entropy (MSE) area under curve (AUC), fast Fourier transform (FFT) low frequency (LF), FFT high frequency (HF), detrended fluctuation analysis (DFA) AUC, and wavelet spectral density AUC measures of variability. In addition, for 3 out of 3 non-infected patients, all measures, except RMSSD, SampEn and MSE, showed no significant reduction. Notably, these measures also showed a substantial correlation (>+30%) amongst each other (Table 5).

**Table 5:** Mean population (n = 17) correlation amongst diverse HRV measures. Correlations greater than +30% are bolded and shown in parenthesis.

	SD	RMSSD	Power Law Slope	Power Law Intercept	SampEn	MSE AUC	FFT LF/HF	FFT LF	FFT HF	DFA AUC	DFA Alpha
**RMSSD**	**(0.61)**										
**Power Law Slope**	0.16	−0.21									
**Power Law Intercept**	**(0.73)**	**(0.31)**	**(0.70)**								
**SampEn**	**(0.45)**	**(0.72)**	−0.22	0.24							
**MSE AUC**	**(0.39)**	**(0.54)**	−0.21	0.23	**(0.87)**						
**FFT LF/HF**	−0.07	−0.41	0.03	−0.02	−0.25	−0.08					
**FFT LF**	**(0.61)**	**(0.70)**	−0.17	**(0.33)**	**(0.60)**	**(0.57)**	−0.02				
**FFT HF**	**(0.50)**	**(0.84)**	−0.15	0.26	(0.58)	(0.48)	−0.43	**(0.79)**			
**DFA AUC**	**(0.80)**	**(0.70)**	−0.14	**(0.55)**	**(0.67)**	**(0.67)**	−0.05	**(0.72)**	**(0.57)**		
**DFA Alpha**	0.30	−0.30	**(0.47)**	**(0.51)**	−0.28	−0.25	0.13	−0.20	−0.23	0.07	
**Wavelet AUC**	**(0.77)**	**(0.72)**	−0.17	**(0.51)**	**(0.69)**	**(0.68)**	−0.08	**(0.72)**	**(0.58)**	**(0.99)**	0.01

**Figure 1:**
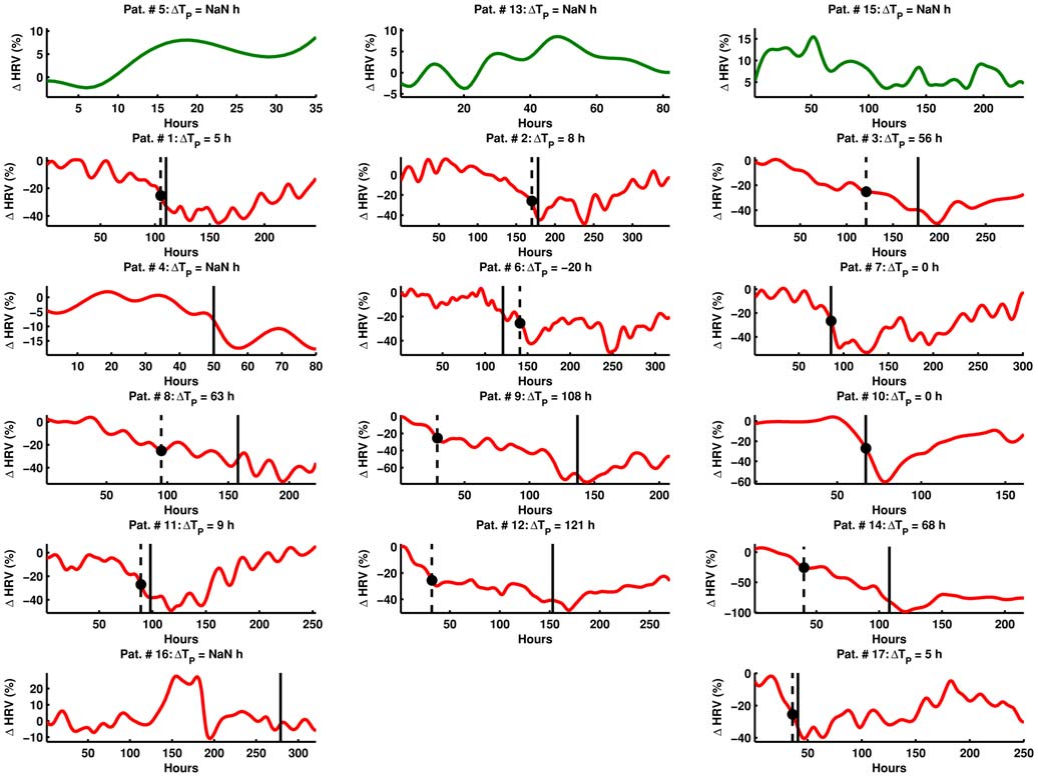
Individual smoothed percentage change. ((HRV) in wavelet variability. Individual wavelet (HRV is studied every 1 h in infected (n = 14) and non-infected (n = 3) patients from 0 h (time after baseline variability) up to the end of the study. Green plots are non-infected patients whereas red plots are infected patients. Solid vertical line denotes the time of sepsis. Lead time (TP is studied for 25% drop (dashed vertical line and dot) from baseline (first 24 h) HRV.

Figure 1 shows the difference in individual patterns of variability for infected (n = 14, red plots) and non-infected (n = 3, green plots) patients for the wavelet measure (AUC) of ΔHRV. In addition, Figure 1 shows the timing of a 25% drop in wavelet ΔHRV (dashed vertical line and dot) in comparison to the time of sepsis (solid vertical line for infected patients) for all patients. Except two patients (Patient #s 4 & 16) out of the 14 infected (n = 14, red plots), all show a 25% drop in ΔHRV in the neighborhood of sepsis. For all non-infected patients (n = 3, green plots), no 25% drop was observed in wavelet ΔHRV. In Figure 1, a positive lead time ΔT_P_ means the 25% drop occurred prior to sepsis, a negative ΔT_P_ means it occurred after sepsis, and ΔT_P_ = NaN (not a number) means no 25% drop occurred. Table 6 summarizes the lead time (ΔT_P_) for all patients (n = 17) studied for 25% drop in ΔHRV for SD, RMSSD, power law intercept, FFT HF, FFT LF, DFA, wavelet, SampEn, and MSE metrics of HRV. We note that except for power law analysis, the 25% drop in ΔHRV occurs prior to sepsis (positive ΔT_P_) for majority of the infected patients.

**Table 6:** Lead time ΔT_P_ (hours) at 25% drop from baseline (first 24 h) HRV.

**Non-infected**									
**ID**	**SD**	**RMSSD**	**Power Law**	**FFT HF**	**FFT LF**	**DFA**	**Wavelet**	**SampEn**	**MSE**
5	NaN	NaN	NaN	NaN	NaN	NaN	NaN	NaN	NaN
13	NaN	−28	NaN	NaN	32	NaN	NaN	−27	−39
15	NaN	NaN	NaN	NaN	NaN	NaN	NaN	NaN	NaN
**Infected**									
**ID**	**SD**	**RMSSD**	**Power Law**	**FFT HF**	**FFT LF**	**DFA**	**Wavelet**	**SampEn**	**MSE**
1	69	72	NaN	96	96	7	5	73	72
2	23	156	NaN	32	177	10	8	53	38
3	108	135	NaN	91	97	93	56	130	116
4	47	NaN	NaN	6	0	NaN	NaN	NaN	5
6	68	82	NaN	NaN	−21	−4	−20	85	84
7	7	85	−11	21	65	2	0	45	65
8	109	110	NaN	68	105	67	63	108	85
9	116	114	18	114	116	109	108	119	118
10	4	7	NaN	9	9	1	0	4	3
11	79	80	NaN	97	97	11	9	82	81
12	138	141	NaN	138	142	122	121	125	127
14	56	80	19	47	79	70	68	78	73
16	227	NaN	NaN	235	235	NaN	NaN	223	226
17	38	39	NaN	15	39	7	5	16	13
**Mean**	77.79	91.75	8.67	74.54	88.29	41.25	35.25	87.77	79.00
**SD**	59.40	42.67	17.04	64.93	69.40	47.41	46.43	56.57	58.47

Observations with NaN excluded for computation of mean and SD.

**Figure 2:**
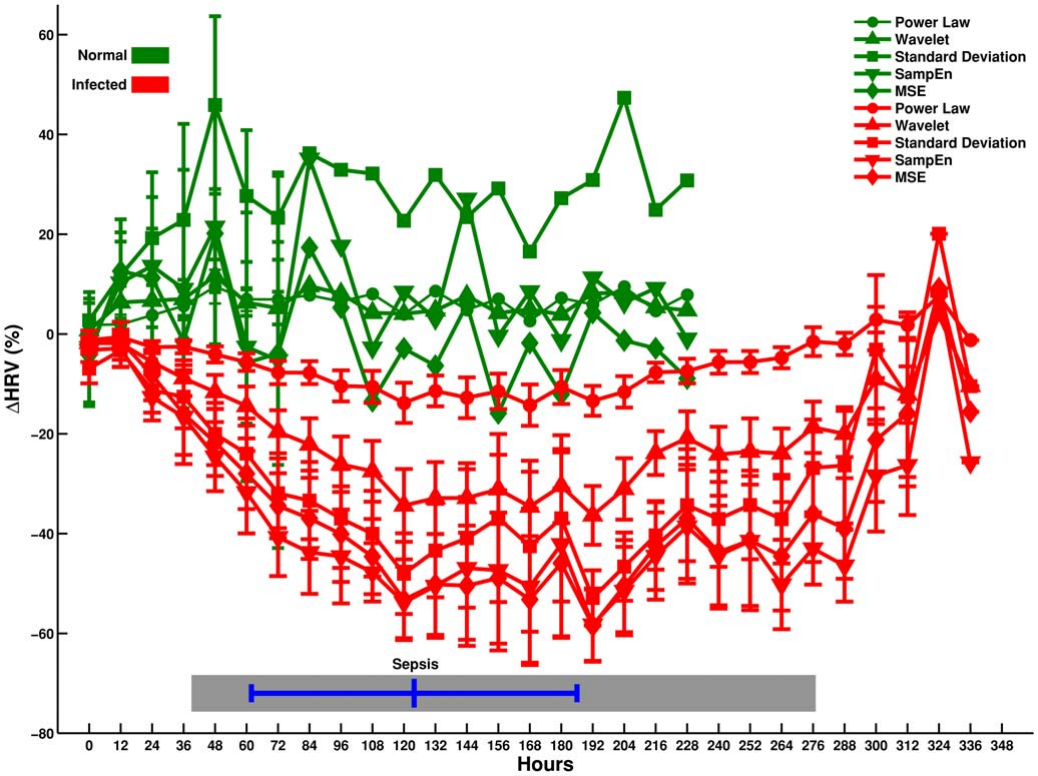
Average percentage change (ΔHRV) in multi-parameter variability. Average multi-parameter (HRV is studied every 12 h in infected (n = 14) and non-infected (n = 3) patients from 0 h (time after baseline variability) up to the end of the study. Green plots are non-infected patients whereas red plots are infected patients. The error bars represent the standard error mean (SEM). The grey horizontal bar at the bottom represents the range of hours where sepsis occurred for all infected patients. The blue horizontal line at the bottom represents the mean time ±standard deviation of sepsis for all infected patients.

**Figure 3:**
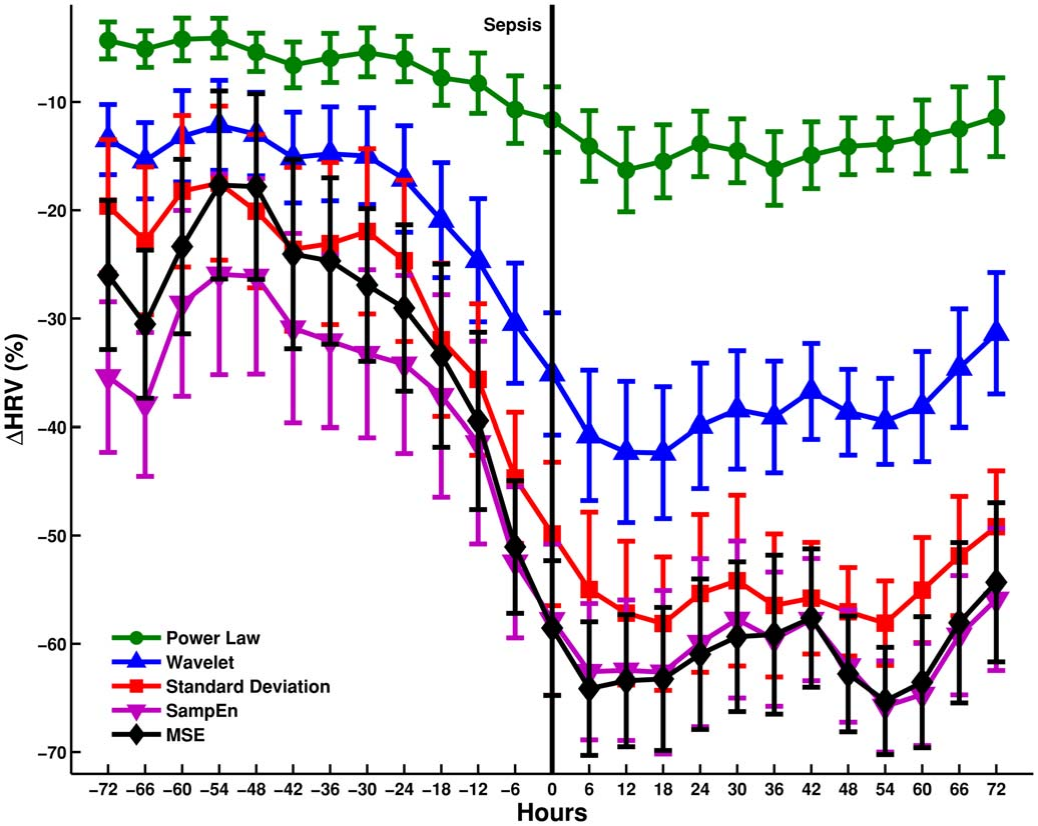
Average percentage change (ΔHRV) in multi-parameter variability around sepsis. Average multi-parameter ΔHRV is studied every 6 h in infected (n = 14) patients±72 h around sepsis (0 h). The error bars represent the standard error mean (SEM).

Figure 2 shows the difference in average patterns of variability every 12 h for the infected (n = 14) and non-infected (n = 3) population for five diverse HRV measures. For these measures, the infected population (plotted in red) shows a distinct and continuous drop in variability from 24–120 h whereas no such drop is observed for the non-infected population (plotted in green). Figure 3 shows the mean variation every 6 h in the above diverse HRV measures zoomed to (72 h around sepsis (time = 0 h) for infected patients (n = 14). Plots in Figure 2 and Figure 3 show a visually evident drop in diverse HRV measures before sepsis; led by entropy measures, followed by standard deviation, wavelet, and power law measures of HRV.

## Discussion

This study represents a pilot investigation of the methods and clinical relevance of tracking HRV continuously in high-risk patients prior to, during, and following clinical diagnosis and treatment for sepsis. We observed a significant loss (defined as a 25% reduction) in multi-parameter HRV in 12/14 or 86% of patients treated for sepsis. None of the patients (0/3) without sepsis demonstrated a significant loss in multi-parameter HRV. Evaluating individual patients as their own control, we observed that the occurrence of loss of HRV was temporally associated with clinical diagnosis and treatment of sepsis in individual patients. The onset of drop of HRV occurred prior to clinical diagnosis and treatment of sepsis, and importantly, recovery of HRV was observed as the patients improved (see Figures 1, 2 & 3). Although this study must be interpreted in light of the small sample size and observational nature, the temporal association demonstrated in this study utilizing continuous variability analysis is in keeping with past investigations utilizing intermittent variability analysis where reduced HRV was demonstrated in the presence of sepsis in adults [11]–[15] and infants [18]–[20].

For infected patients, we note that ΔT_P_ values greater than 120 h are probably “false alarms” where the 25% drop does occur momentarily but is not followed by further sustained drops in ΔHRV. For example for Patient #16, the standard deviation metric shows a ΔT_P_ of 237 h (Table 6). However, the variability pattern of standard deviation (not shown) is very similar to that of the wavelet metric for Patient #16 (Figure 1). This means that such drops in ΔHRV cannot be used for prediction of sepsis with appropriate confidence levels. That is, for infected patients, the drop in ΔHRV should be continuous and progressive for the prediction of sepsis to have more reliability and robustness. We found that amongst all metrics studied for prediction of sepsis, the wavelet analysis showed the highest reliability and stability whereby ΔT_P_ was within a reasonable timeframe of sepsis, and “false alarms” were minimal with regard to both infected and non-infected patients (Figure 1 and Table 6). The main reason for the success of wavelet analysis seems to be the fact that it is a recursive filtering technique which is capable of analyzing a signal in both time and frequency domains simultaneously. These attributes make the wavelet analysis robust to both noise and non-stationarity in the data, thus allowing it to characterize signal variability in the most efficient and accurate manner.

While other studies have clearly documented population-based changes in HRV in association with sepsis, this study identified individual patient loss of HRV equal to 25% in association with, and prior to, clinical diagnosis and treatment of sepsis in all but two patients. Of note, the patient (Patient #16, Figure 1) who showed no drop in HRV but was diagnosed and treated for sepsis was a patient with insulin dependent diabetes. This finding requires further study, in particular since existing literature suggests that HRV may not be an effective tool for characterizing the autonomic nervous system control in patients with diabetes [23]–[24].

Although this investigation supports the feasibility of long term HR monitoring (up to 16 days) along with continuous HRV analysis in non-stationary HR data sets in ambulatory patients, the use of Holter monitors with gel electrodes proved demanding for patients recovering from BMT. A total of 4 patients out of 21 (19%) dropped out prematurely after initiating the study. For the 17 patients who completed the study, the Holter data lost due to non-compliance to monitoring was 6%. We note that some of the reasons for non-compliance were beyond the control of the patients. For example, if a patient had to bathe or was in discomfort, the Holter had to be taken off and sometimes a particular schedule for arrival at the hospital (resulting in delay in new Holter attachment and data loss) was based on bed availability, physician availability, and other similar factors. The quality of the Holter data recorded was clean and satisfactory; the data lost to RR cleaning was only 1.06 days (0.6%) in a total of 176 days of RR-interval data. We also visually inspected the recorded RR-interval data and found it to be satisfactory. We encountered difficulty in motivating patients to accurately and punctually maintain a diary of clinical events. This precluded us from rigorously correlating our HRV measures with other clinical data such as periodic temperature measurements, symptoms such as frequency ranges of diarrhea and vomiting, etc.

In 2 out of the 3 normal patients, relatively short Holter monitoring periods of 3 and 5 days, were observed. This was because these patients withdrew from the study after these timeframes. However, these patients did not develop sepsis during the monitoring period. Thus, they were considered as uninfected patients for the study.

In order to visually observe and computationally detect changes in HRV over time, we have pioneered a process of double temporal integration. The first step of this process involves computing a moving window based change in HRV over time. The second step involves smoothing of the change in HRV over time utilizing the moving average and data interpolation techniques. We observed that this process was instrumental in highlighting and studying stable and progressive changes in HRV hours prior to the onset of sepsis.

In addition to the general process of continuous variability analysis, we had to evolve the process of continuous individualized monitoring of individual variability metrics, where no guidelines exist. For example, in the performance of iteratively repeated sample and multi-scale entropy analysis, it remains an unanswered question as to whether the tolerance r, calculated as 15% of the standard deviation (SD), should be fixed, or continuously re-calculated based on the changing SD for every window instance. We empirically observed marked reduction in both entropy metrics by keeping the tolerance r fixed as 15% of the SD of the first 24 h (baseline) of HR data [r = 0.15*SD(HR_baseline_)] as patients developed sepsis. However, re-calculating the entropy metrics based upon a variable value for r (a new r based on SD of each window instance), the reduction in entropy was not evident. This highlights the inter-dependence of the entropy metrics and the time-domain SD metric and requires further theoretical and empirical study.

We also observed that the DFA AUC and wavelet spectral density AUC metrics showed identical HRV patterns when computed continuously over time (99% correlation between DFA AUC and wavelet spectral density AUC – see Table 5). Our empirical observation and the correlation analysis highlight the interdependence between multiple measures of HRV, and the unresolved debate regarding the most efficient and effective means to characterize HRV.

The pathophysiology of altered HRV still remains uncertain, and was not the focus of this study. The loss of overall variation and complexity metrics are in keeping with existing hypotheses regarding augmentation of sympathetic tone, “decomplexification” of physiologic signals [25], as well as “uncoupling of biologic oscillators” [26] that have been proposed to occur in association with sepsis and critical illness. Regardless of the possible mechanisms, we interpret the alteration in HRV occurring in association with sepsis as reflective of a fundamental alteration to the underlying complex system producing the dynamics.

Although this study was not designed for a comprehensive lead-time analysis, we observed changes in HRV at least 24 h prior to the clinical diagnosis and treatment of sepsis. This analysis was limited by the fact that we were unable to identify the precise time of onset of sepsis, and rather identified the onset of antibiotic treatment as a temporal point for comparing the results of the variability analyses. Half of the patients were diagnosed with sepsis based upon detection of a fever 60–90 minutes prior to administration of antibiotics. Thus, intermittent sampling of temperature (about every 6 h) may have delayed diagnosis by several hours. Of note, we investigated the technology to monitor skin temperature continuously, and found it imprecise and unreliable. However, experimental models of sepsis have noted changes in HRV at least 6 h prior to the onset of fever [27]. Following these results, it will be worthwhile to correlate the diverse measures of HRV with circulating biomarkers of sepsis and inflammation. Our study group comprised ambulatory outpatients recovering from myeloablative therapy, who were already encumbered by continuous HR monitoring, and frequent blood sampling was not perceived as ethical in absence of these data.

Numerous unanswered questions remain. Although the clinical significance of this investigation lies in its logical extension, namely the potential of utilizing a system of prospective continuous HRV monitoring to provide an early warning system for sepsis, a larger validation cohort is clearly required, along with definitive investigations regarding generalizability, sensitivity and specificity. It is entirely unclear if hospitalized patients (e.g. surgical or critically ill patients) will demonstrate such profound loss of HRV in association with new onset of sepsis. Although a panel of HRV metrics has been recommended to optimally characterize variability, it remains to be seen which offer the optimal means to herald the presence of sepsis. Last, a more in depth evaluation of lead-time offered by HRV monitoring is warranted. As evident above from the discussion regarding double temporal integration, a process of prospective continuous HRV monitoring will necessarily lead to a delay inherent to the interval of time necessary to characterize variability and thus the underlying system. Akin to the uncertainty principle, there is a balance between the need for an adequate interval of time to characterize the system, and the need to characterize the system at any point in time. Optimization of this balance may depend on clinical applications. Nonetheless, the promise of a novel dimension of monitoring based on continuous variability analysis is supported by this observational trial, and merits the attention of future investigations.
